# Communication and Relational Ties in Inter-Professional Teams in Norwegian Specialized Health Care: A Multicentre Study of Relational Coordination

**DOI:** 10.5334/ijic.3432

**Published:** 2018-04-27

**Authors:** Merethe Hustoft, Øystein Hetlevik, Jӧrg Aßmus, Sverre Størkson, Sturla Gjesdal, Eva Biringer

**Affiliations:** 1 Centre for Habilitation and Rehabilitation in Western Norway, Haukeland University Hospital, Department of Global Health and Primary Health Care, University of Bergen, NO; 2 Department of Global Health and Primary Health Care, University of Bergen, NO; 3 Centre for Clinical Research, Haukeland University Hospital, NO; 4 Health West IKT, Research Network on Integrated Health Care, Helse Fonna Local Health Authority, NO; 5 Research Network on Integrated Health Care, Helse Fonna Local Health Authority, Section of Research and Innovation, Helse Fonna Local Health Authority, NO

**Keywords:** Teamwork, integrated care, Relational Coordination, coordination, multilevel analysis

## Abstract

**Introduction:**

The delivery of integrated care depends on the quality of communication and relationships among health-care professionals in inter-professional teams. The main aim of this study was to investigate individual and team communication and relational ties of teams in specific care processes within specialized health care.

**Methods:**

This cross-sectional multi-centre study used data from six somatic hospitals and six psychiatric units (N = 263 [response rate, 52%], 23 care processes) using a Norwegian version of the Relational Coordination Survey. We employed linear mixed-effect regression models and one-way analyses of variance.

**Results:**

The mean (standard deviation) relational coordination total score ranged from 4.5 (0.33) to 2.7 (0.50). The communication and relationship sub-scale scores were significantly higher within similar functional groups than between contrasting functional groups (*P* < .05). Written clinical procedures were significantly associated with higher communication scores (*P* < .05). The proportion of women in a team was associated with higher communication and relationship scores (*P* < .05).

**Conclusion:**

The Relational Coordination Survey shows a marked variation in team functions within inter-professional teams in specialized health-care settings. Further research is needed to determine the reasons for these variations.

## Background

Many patients today are in contact with multiple health-care services and professionals. This is a result of the complexity of modern health care and the high prevalence of patients with chronic diseases and multiple co-morbidities [1,2,3,4,5,6].

The implementation of inter-professional teams in health care began in the 1970s. It underwent resurgence in the late 1980s because evidence suggested that improved integrated care and coordination could save lives [7]. Studies have found that the quality of patient care depends on skilled professionals collaborating in teams [8,9,10,11]. Teamwork is considered paramount for the coordination of integrated care in specialized health-care settings [5,11,12,13]. With inter-professional teamwork, treatment plans become more complementary to patient needs and care becomes more efficient [3,14,15,16]. Deneckere et al. [6] identified numerous individual and team characteristics that influence teamwork, such as conflict management, communication skills, frequency of meetings, common goals, team size, composition, and leadership [6].

Research has found that the quality of communication among health-care professionals and quality of their underlying relationships are central aspects of team functioning [5,6,17,18,19,20]. However, few studies have investigated the association of individual and team-level characteristics with team function [6,8,21]. Studies by Mickan [8] and Vinokur-Kaplan [21] have identified team composition, shared objectives, and team size as important predictors of team functioning. Smaller teams with greater occupational diversity are associated with higher overall effectiveness [6,19].

Deneckere et al. [6] found that teams that develop clinical procedures showed better inter-professional teamwork and a higher level of organized care. Further, the authors also identified a significant increase in the level of individual competence and perceived “teamness” as a result of implementing clinical procedures. However, Deneckere et al. did not observe any association between communication skills or relational ties in inter-professional teams and the implementation of clinical procedures [6].

Research has produced inconsistent results with regard to the effect of team composition and size on teamwork. No investigations have assessed the relationship among age, use and development of clinical procedures, level of experience, and team functioning.

Owing to the lack of reports assessing the association between team function and relevant individual and team factors, we conducted a study on a range of inter-professional teams to determine the associations among age, use and development of clinical procedures, composition, years of experience in team, team size, and team functioning at both the individual and team level. In the present study, we thus undertook the following. First, within specialized health-care settings, we investigated levels of communication and relational ties in inter-professional teams in specific care processes. Second, we assessed the association between individual- and team-level characteristics of inter-professional teams and communication and relational ties in those teams.

## Theory and Methods

### Relational Coordination

A recent review identified 10 measurement tools measuring teamwork that meet the criteria for psychometric validity [20]. Those tools survey teamwork functions, such as communication, coordination, shared decision making, collaboration, active conflict management, shared objectives and respect. The Relational Coordination Survey was among the 10 recommended measurement tools [8]. This survey is used within health-care services as well as in primary care, community, and hospital settings; it is a useful tool when measuring the quality of communication and relational ties in inter-professional teams in different parts of health care [22,23,24,25,26].

Relational coordination is defined as a “mutually reinforcing process of interaction between communication and relationships carried out for the purpose of task integration” [27]. Rather than examining relationships among particular individuals, the focus of relational coordination is on relationships among professional groups with similar roles (hereafter, “functional groups”) [8]. Relational coordination theory has been found to be a sound framework for investigating care coordination in inter-professional teams [28].

The original Relational Coordination Survey has two sub-scales: a communication sub-scale comprising four survey questions (evaluating the frequency, accuracy, timeliness, and problem-solving nature of communication); and a relationship sub-scale with three survey questions (concerning shared goals, shared knowledge, and mutual respect) [25]. The seven items in the Relational Coordination Survey employ a five-point Likert scale. Respondents are asked to complete each item according to their perception of communication or relationships with specific functional groups of health professionals in their team, e.g., physicians, nurses, physiotherapists, and administrative personnel. This creates a matrix with seven Relational Coordination Survey items for each functional group.

The functional groups included in each team vary according to which types of functional groups are considered relevant for the particular care process under assessment. The scores for the two sub-scales are derived by calculating the mean of the four communication and three relationship scores [29]. Higher scores indicate better communication and relational ties within the inter-professional team.

For use in the present study, the Relational Coordination Survey was translated to Norwegian and piloted on 10 health-care professionals within a hospital by Størkson et al. [30]. An authorized translation agency translated the US-English version of the Relational Coordination Survey into Norwegian language. A research team discussed linguistic and cultural aspects. Minor amendments on the Norwegian Relational Coordination Survey were made due to minor difficulties regarding the interpretation of items and contextual issues before a second authorized translator translated the survey back into English language. This version was found comparable to the original version. This was accepted by the author of the original version of Relational Coordination Survey. A psychometric assessment of the Norwegian version of the Relational Coordination Survey constitutes part of the present study.

### Design and participants

This cross-sectional multi-centre study used data from six somatic hospitals and six specialist psychiatric units within the Western Norway Regional Health Authority, constituting 27 care processes in total. The team members (N = 503) received information about the project by e-mail, including a link to the Relational Coordination Survey in Corporater Surveyor, version 3.3 (Corporater Inc., Norway) [30]. In all, 301 health-care professionals (60%) responded. All these participants were used in analyses of the psychometric properties of the Norwegian version of the Relational Coordination Survey.

We inspected the data for inconsistencies and missing items. Respondents with missing items were excluded as follows. First, we excluded individuals who had completed less than 40% of the survey response alternatives (there was one response alternative for each functional group of health professionals) among each of the seven items. Second, we excluded participants if they responded to three or fewer of the seven items. Finally, we excluded respondents in teams with fewer than four valid respondents. That left 263 (52%) participants in the final analysis, representing 23 care processes (Table 1).

**Table 1:** Overview of team characteristics in 23 care processes included in the valid sample, team size, fraction of women in team, age and professional group distribution and clinical procedure use in teams (N = 263).

Care process	Team size	Valid responses		Age group		Functional group			Clinical procedure

				≤39	≥40	Reg. Nurse (somatic)	Physician	Therapy/other	In team

	N	N (%)	N (%)	N (%)	N (%)	N (%)	N (%)	N (%)	Yes/No

1. ADHD 1^1^	10	4 (40%)	3 (75%)	2 (50%)	2 (50%)	1 (25%)	0 (0%)	3 (75%)	Yes
2. ADHD 2^2^	33	18 (54%)	13 (72%)	10 (56%)	8 (44%)	3 (17%)	6 (33%)	8 (44%)	Yes
3. Hip arthroplasty	9	5 (56%)	4 (80%)	4 (80%)	1 (20%)	2 (40%)	1 (20%)	2 (40%)	Yes
4. Acute stroke	19	16 (79%)	13 (81%)	8 (50%)	8 (50%)	8 (50%)	4 (25%)	4 (25%)	Yes
5. Cerebral palsy, children	14	11 (79%)	11 (100%)	1 (9%)	10 (91%)	0 (0%)	0 (0%)	11 (100%)	Yes
6. Sinus surgery	19	13 (68%)	7 (54%)	5 (38%)	5 (38%)	4 (31%)	7 (54%)	2 (15%)	No
7. Diabetes treatment, children	18	12 (67%)	11 (92%)	3 (23%)	9 (75%)	9 (75%)	2 (17%)	1 (8%)	Yes
8. VT, diagnostic process and treatment^3^	16	16 (100%)	10 (63%)	11 (69%)	4 (25%)	10 (63%)	4 (25%)	0 (0%)	Yes
9. Elective hip surgery	20	16 (80%)	12 (75%)	4 (25%)	12 (75%)	9 (56%)	5 (31%)	2 (13%)	Yes
10. Stroke	10	5 (50%)	4 (80%)	2 (40%)	3 (60%)	1 (20%)	0 (0%)	4 (80%)	Yes
11. In vitro fertilisation	17	13 (77%)	11 (85%)	2 (15%)	11 (85%)	4 (31%)	2 (15%)	7 (54%)	Yes
12. Knee arthroplasty	15	9 (60%)	7 (78%)	2 (22%)	6 (67%)	5 (56%)	0 (0%)	4 (44%)	Yes
13. Chronic Obstructive Pulmonary Disease	26	15 (58%)	10 (67%)	11(73%)	4 (27%)	8 (53%)	5 (34%)	2 (13%)	No
14. Lung cancer- diagnostic process	21	10 (48%)	6 (60%)	7 (70%)	3 (30%)	4 (40%)	5 (50%)	1 (10%)	No
15. Breast cancer surgery	14	7 (50%)	7 (100%)	3 (43%)	4 (57%)	5 (71%)	0 (0%)	2 (29%)	Yes
16. Tonsillectomy/adenotomy, children	15	10 (67%)	6 (60%)	3 (30%)	7 (70%)	4 (40%)	3 (30%)	2 (20%)	Yes
17. Arthroscopy knee, meniscus surgery	25	15 (60%)	5 (33%)	8 (53%)	7 (47%)	5 (34%)	8 (53%)	2 (13%)	Yes
18. Psychosis (planned admission)	18	9 (50%)	8 (89%)	5 (56%)	4 (44%)	0 (0%)	8 (89%)	1 (11%)	Yes
19. Psychosis (outpatient)	14	9 (64%)	5 (56%)	4 (44%)	5 (56%)	0 (0%)	6 (67%)	3 (33%)	Yes
20. Psychosis	24	13 (54%)	9 (69%)	4 (31%)	9 (69%)	1 (8%)	10 (77%)	2 (15%)	Yes
21. Stroke rehabilitation	26	12 (46%)	10 (83%)	7 (58%)	5 (42%)	4 (33%)	3 (25%)	5 (42%)	Yes
22. Tonsillectomy, adult	15	8 (53%)	5 (63%)	4 (50%)	4 (50%)	2 (25%)	5 (63%)	0 (0%)	Yes
23. Respiratory diseases, emergency department	22	17 (77%)	8 (47%)	9 (53%)	8 (47%)	9 (53%)	4 (24%)	2 (12%)	Yes
24. **Total**		**263 (52%)**	**185 (70%)**	**119 (45%)**	**142 (54%)**	**98 (37%)**	**88 (33%)**	**70 (27%)**	

^1^ Attention-Deficit/Hyperactivity Disorder, diagnostic process 1. ^2^ Attention-Deficit/Hyperactivity Disorder, diagnostic process 2. ^3^ Venous thrombosis, diagnostic process and treatment.

### Individual-specific variables

Respondents were asked to report the following information: professional group (registered nurse [somatic], physician, medical laboratory technician, physiotherapist, social worker/occupational therapist/social educator, or administrator/coordinator/advisor), sex, age group (20–29, 30–39, 40–49, 50–59, or 60–69 years), and whether they used a written clinical procedure in their daily care of the patient group (no, under development, or in use). We dichotomized age (≤39 versus ≥40 years), use of clinical procedures (no versus yes/under development), and profession (not physician versus physician).

### Team-specific variables

Based on the individual variables, we defined team variables to characterize the composition of the team: the proportions of (1) women; (2) team members older than 40 years; and (3) physicians in the team and team size. The team was said to have a clinical procedure if ≥80% of team members answered yes or under development to the related question.

### Predictor variables

Individual-specific predictor variables for the survey communication and relationship sub-scales, as reported by each professional respondent, were age, sex, use of clinical procedures, and physician in the team. Team-specific predictor variables for the survey sub-scales (summarized for each team) were proportion of women in team, team members >40 years, use of clinical procedures, proportion of physicians, and team size.

## Statistical analysis

We employed confirmatory factor analysis (maximum likelihood estimation with robust standard errors, Satorra-Bentler correction) to test the factor structure. To define a satisfactory model fit, we used the following: a cut-off at 0.95 or higher for the comparative fit index; cut-off at < 0.06 to 0.08 for the root mean square error of approximation; cut-off at 0.8 or lower for the standardized root mean square residual; and cut-off at 0.95 or higher for the Tucker-Lewis index [31]. To assess intra-scale consistency, we computed Cronbach’s alpha. A construct validity test could not be performed as there were no comparative instruments available for Norwegian health care settings.

We tested differences among functional groups (nurses, physicians, therapists/other) with regard to the communication and relationship sub-scale scores by one-way analysis of variance and illustrated by graphical tools. To assess the association between the predictor variables and the sub-scale scores, we used linear regression models with the communication and relationship sub-scale scores as outcome variables.

For the individual variables (age, sex, profession, and use of clinical procedures), we took into account correlations within each team. Thus, we used a linear mixed-effects model, including the individual variables as fixed factors and team affiliation as random effect.

For the team-specific variables, we used a simple linear regression model with the team mean of the sub-scales as outcome and team-specific variables as predictor. We estimated the univariate model for each predictor as well as the multivariate model for the individual variables and team-specific variables. Tests were two-tailed and the significance level was set to 0.05.

The computation was done in SPSS 23 (IBM Corp., Armonk, NY) and R 3.3 [32] with the packages lavaan 0.5 (confirmatory factor analyses) [33] and nlme 3.1 (linear mixed-effect model) [34]. The graphics were produced using Matlab 9.0 (The Mathworks Inc., Natick, MA).

Informed consent to participate was assumed when respondents returned a completed survey. Returned questionnaires were de-identified and data were stored according to appropriate regulations. This study was approved by the Norwegian Social Science Data Services in 2012 (reference no. 29128), which, with this type of material, is the relevant body for approval.

## Results

### Psychometric properties

Previous research has suggested both a one-factor and two-factor approach for the Relational Coordination Survey [8,35]. However, the factor structure of our sample revealed a better model fit with the two-factor structure than the one-factor model [8,35]. Three estimates of fit—comparative fit index, Tucker-Lewis index, and standardized root mean square residual (the latter is independent of the χ² and sample size [32])—showed: 0.86, 0.79 and 0.09 for the 1-factor solution, respectively. Further, the chi-square from the 1-factor solution was 164.8 (p =< 0.001) with 14 degrees of freedom giving a normed χ² of 11.8. For the 2-factor solution the three estimates of fit showed an acceptable fit: 0.93, 0.89, and 0.06, respectively. Further, the chi-square from the 2-factor solution was 84.2 (p =< 0.001) with 13 degrees of freedom giving a normed χ² of 6.48. A chi-square difference test (χ²_diff_ = 83.6, p =< 0.001) suggested that fit was most favourable the 2-factor solution.

Cronbach’s alpha for the communication and relationship sub-scales was 0.93 and 0.80, respectively. This estimated intra-scale consistency supported the internal reliability of the measured items in a two-factor structure [36].

### Individual-level associations

Table 2 lists the reported survey scores in each professional’s team by different individual characteristics. Among the mean scores in Table 2, there is a trend for higher scores in the relationship than with the communication sub-scale. There are, however, no clear age or sex-related differences.

**Table 2:** Relational Coordination Survey mean (standard deviation) communication and relationship subscale scores according to respondent’s functional group, sex, age group, and use of clinical procedures in 23 care processes (N = 263).

Predictor variables	Communication	Relationship

**Functional Group**		

Registered nurse (somatic)	3.3 (0.67)	3.7 (0.60)
Physician	3.4 (0.78)	3.8 (0.61)
Therapy/others	3.6 (0.63)	3.8 (0.61)
**Sex**		

Male	3.3 (0.72)	3.9 (0.56)
Female	3.5 (0.66)	3.8 (0.62)
**Age group**		

≤39	3.3 (0.72)	3.7 (0.64)
40–49	3.4 (0.69)	3.8 (0.54)
≥50	3.5 (0.71)	3.8 (0.61)
**Clinical procedure**		

No	3.1 (0.65)	3.6 (0.53)
Under development	3.6 (0.59)	3.8 (0.44)
In use	3.4 (0.72)	3.9 (0.64)

**Figure 1:**
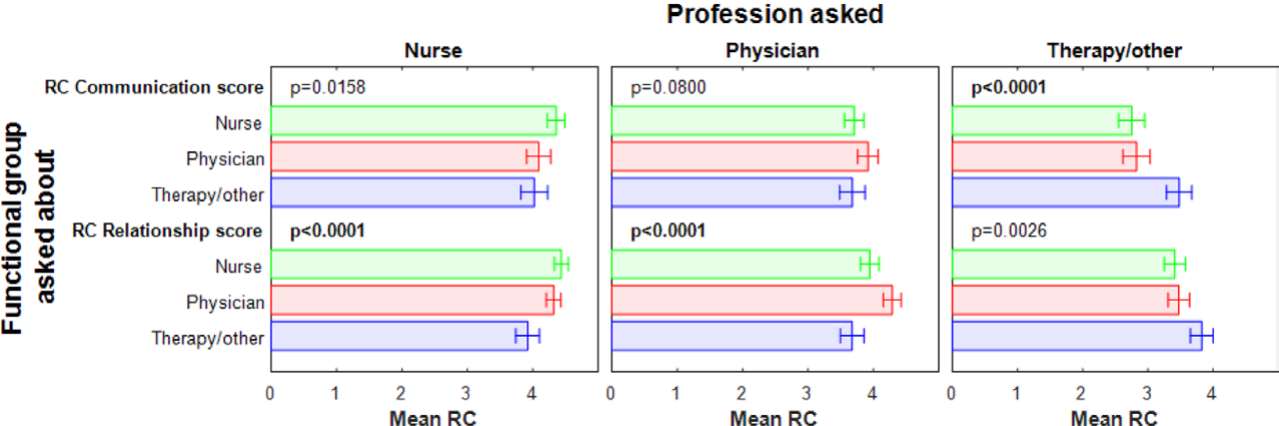
Relational Coordination Survey communication and relationship sub-scale scores within and between unique functional groups (N = 263).

Communication sub-scale scores were significantly higher within unique functional groups than between contrasting functional groups (Figure 1): nurses and nurses, 4.4 (95% confidence interval, 4.22–4.27, *P* = 0.016); physicians and physicians, 3.9 (95% confidence interval, 3.76–4.07, not significant); and therapy/others and therapy/others, 3.5 (95% confidence interval, 3.29–3.67, *P* = 0.001). The relationship sub-scale scores were as follows: nurses and nurses, 4.4 (95% confidence interval, 4.33–4.55, *P* = 0.001); physicians and physicians, 4.3 (95% confidence interval, 4.15–4.42, *P* = 0.001); and therapy/others and therapy/others, 3.8 (95% confidence interval, 3.65–4.00, *P* = 0.003).

Individual team members who reported that they used a written clinical procedure on a daily basis or were in the process of developing procedures reported higher communication sub-scale scores than team members who did not use or were not developing such a procedure (Table 2). Table 3 indicates that using or developing a clinical procedure was significantly associated with higher communication sub-scale scores in the multivariate model (B = 0.20; 95% confidence interval for B, 0.00–0.41; *P* = 0.049). There was a marginal non-significant result that being a physician was associated with higher relationship sub-scale scores (B = 0.17; 95% confidence interval, 0.00–0.34; *P* = 0.051).

Table 3Individual-level characteristics’ associations with Relational Coordination Survey communication and relationship subscale scores (N = 263).

**Table:** 

Communication sub-scale scores	Univariate			Multivariate		

	Estimates			Estimates		

Individual predictors^1^	B	95%CI	p-value	B	95%CI	p-value

Age (>= 40)^2^	0.05	(–0.09, 0.19)	0.479	0.05	(–0.09, 0.19)	0.505
Sex^3^	0.09	(–0.06, 0.24)	0.228	0.12	(–0.06, 0.29)	0.188
Use of clinical procedures^4^	0.18	(–0.02, 0.37)	0.081	0.20	(0.00, 0.41)	0.049
Physician^5^	0.01	(–0.14, 0.17)	0.858	0.14	(–0.04, 0.32)	0.130

**Table:** 

Relationship sub-scale scores	Univariate			Multivariate		

	Estimates			Estimates		

Individual predictors^1^	B	95%CI	p-value	B	95%CI	p-value

Age (>= 40)^2^	0.04	(–0.09, 0.18)	0.533	0.06	(–0.08, 0.20)	0.407
Sex^3^	–0.17	(–0.32, –0.03)	0.019	–0.10	(–0.26, 0.07)	0.259
Use of clinical procedures^4^	0.09	(–0.09, 0.28)	0.328	0.11	(–0.08, 0.30)	0.269
Physician^5^	0.18	(0.03, 0.33)	0.016	0.17	(0.00, 0.34)	0.051

^1^ Linear Mixed Effects Model, individual, random effect: team. ^2^ Reference category; age group ≤39. ^3^ Reference category; men. ^4^ Reference category; no clinical procedure in place. ^5^ Reference category; all other functional groups.

### Team-level associations

With the survey scores for different teams (Table 4), we found the mean (standard deviation) for communication and relationship sub-scale scores ranged from 4.3 (0.52) to 2.7 (0.34) and 4.5 (0.33) to 3.2 (0.71), respectively. Notably, the communication and relationship sub-scale means were among the highest in teams responsible for stroke patients. We found no clear differences concerning in- or outpatient or somatic or psychiatric care processes.

**Table 4:** Means (standard deviations) for Relational Coordination Survey communication and relationship sub-scale scores among 23 care processes included in the valid sample (N = 263).

Care process	Communication	Relationship

Acute stroke	4.3 (0.52)	4.2 (0.52)
In vitro fertilization	4.3 (0.34)	4.5 (0.33)
Stroke treatment	4.2 (0.47)	4.0 (0.46)
Stroke rehabilitation	4.2 (0.45)	4.3 (0.49)
Hip fracture	4.0 (0.53)	4.5 (0.21)
Psychosis (outpatient)	3.8 (0.62)	3.8 (0.56)
Psychosis (planned admission)	3.8 (0.51)	3.9 (0.39)
Cerebral palsy, children	3.8 (0.48)	3.8 (0.49)
Attention-Deficit/Hyperactivity Disorder, diagnostic process 2	3.5 (0.36)	4.1 (0.50)
Knee arthroplasty	3.3 (0.69)	3.4 (0.66)
Hip arthroplasty	3.3 (0.55)	3.9 (0.63)
Tonsillectomy/adenotomy, children	3.3 (0.35)	3.7 (0.35)
Psychosis	3.2 (0.72)	3.3 (0.60)
Breast cancer surgery	3.2 (0.67)	3.5 (0.71)
Chronic Obstructive Pulmonary Disease	3.2 (0.45)	3.7 (0.37)
Diabetes treatment, children	3.2 (0.43)	3.7 (0.24)
Attention-Deficit/Hyperactivity Disorder, diagnostic process 1	3.1 (0.36)	3.9 (0.21)
Tonsillectomy, adult	3.0 (0.75)	3.6 (0.39)
Sinus surgery	3.0 (0.55)	3.6 (0.36)
Arthroscopy knee, meniscus surgery	2.9 (0.76)	3.7 (0.57)
Lung cancer- diagnostic process	2.9 (0.55)	3.6 (0.53)
Respiratory diseases, emergency department	2.7 (0.50)	3.2 (0.71)
Venous thrombosis, diagnostic process and treatment	2.7 (0.34)	3.3 (0.65)

The proportion of women in a team was associated with higher communication and relationship sub-scale scores in the univariate model (respectively, B = 1.68; 95% confidence interval, 0.51–2.85; *P* = 0.007) and (B = 0.99; 95% confidence interval, 0.12–1.85; *P* = 0.028; Table 5).

Table 5Team-level characteristics’ associations between the valid sample of 23 inter-professional teams and Relational Coordination Survey communication and relationship sub-scale scores.

**Table:** 

Communication sub-scale scores	Univariate			Multivariate		

	Estimates			Estimates		

Team specific predictors^1^	B	95%CI	p-value	B	95%CI	p-value

Proportion of women^2^	1.68	(0.51, 2.85)	0.007	2.37	(–0.10, 4.83)	0.059
Proportion of team members older than 40^3^	0.46	(–0.76, 1.67)	0.445	0.01	(–1.42, 1.45)	0.984
Use of clinical procedures^4^	0.21	(–0.89, 1.31)	0.694	0.34	(–0.95, 1.63)	0.579
Proportion of physicians in the team^5^	–0.32	(–1.21, 0.56)	0.460	0.81	(–0.90, 2.53)	0.323
Team size^6^	–0.02	(–0.05, 0.02)	0.416	–	–	–

**Table:** 

Relationship sub-scale scores	Univariate			Multivariate		

	Estimates			Estimates		

Team specific predictors^1^	B	95%CI	p-value	B	95%CI	p-value

Proportion of women^2^	0.99	(0.12, 1.85)	0.028	1.45	(–0.41, 3.31)	0.115
Proportion of team members older than 40^3^	–0.05	(–0.91, 0.81)	0.912	–0.31	(–1.39, 0.77)	0.550
Use of clinical procedures^4^	0.53	(–0.24, 1.29)	0.167	0.58	(–0.39,1 .55)	0.219
Proportion of physicians in the team^5^	–0.25	(–0.86, 0.37)	0.418	0.57	(–0.72, 1.86)	0.359
Team size^6^	–0.01	(–0.03, 0.02)	0.678	–	–	–

^1^ Linear Regression Model, for team means. ^2^ Number of women in team/total number of team members. ^3^ Number of team members ≥40 years of age/total number of team member. ^4^ Reference category; no clinical procedure. ^5^ Number of physicians in team/total number of team members. ^6^ Total number of valid responses in the care process.

## Discussion

Based on the normed χ², comparative fit index, Tucker-Lewis index, and standard root mean square residual estimates of fit from the confirmatory factor analysis; we conclude that the Norwegian version of the Relational Coordination Survey is acceptable for use in specialized health-care settings employing the two suggested sub-scales of communication and relationship. The chi-square test is perceived inappropriate as it is sensitive to large study populations (above 200) and therefore tends to reject models too often [37]. This conclusion is supported by earlier investigations of the factor structure of the survey employing exploratory factor analyses [8,35].

The use of the Relational Coordination Survey in the included care processes revealed relatively large differences in the quality of teamwork through the survey sub-scales (Table 4). The better communication and relational ties in these inter-professional teams may reflect an increased effort to improve integrated care for these patient groups. Previous research has shown that implementation of specific inter-professional teams and specific guidelines within stroke rehabilitation have improved patient outcomes [38,39].

At the level of the individual respondent, we observed that being a physician was associated with higher relationship sub-scale scores within teams. This may reflect physicians typically having a central, coordinating role in inter-professional teams in specialized health-care settings in Norway. However, this result is contrary to that of Hartgerink et al. [23]; they found that being a physician was associated with lower perceived team communication and relational ties. The authors explained this negative association as the result of medical specialists often making their treatment decisions independently of others—and consequently not interacting frequently with other team members.

In the present study, team members in the same profession communicated better with others in the functional group to which they belonged than with members of other functional groups. Inter-professional teamwork has received much attention lately; however, this result may reflect a lack of understanding of different roles and poor communication skills across contrasting functional groups. Furthermore, inter-professional education that includes hands-on inter-professional teamwork practice is not yet fully implemented in all education programmes within health care [40].

Individual team members’ development or daily use of a written clinical procedure was associated with significantly higher communication sub-scale scores (Table 4). This finding may reflect the fact that clinical procedures serve as a coordinating mechanism, assuring necessary levels of communication in inter-professional teamwork [41]. Moreover, Deneckere et al. [6] found that coordination of care and communication improved in inter-professional teams developing clinical procedures. In our study, each team member stated whether or not a written clinical procedure was in daily use. However, the team members reported this information inconsistently. This discrepancy may be explained by respondents’ interpretation of the term “clinical procedure”: it may be understood differently from one respondent to another [30]; it may also reflect individual respondents being unaware of the existence of a particular procedure. Further, respondents who were aware of the existence of the clinical procedure may not actually have used it in the care process.

A team with a greater proportion of female members was associated with higher communication sub-scale scores. One explanation for this finding may be that women tend to be more oriented towards interpersonal relations and social interactions—and therefore provide higher communication sub-scale scores—than men [42]. Research has shown that nurses were more positive towards collaborating in a team environment than physicians, who traditionally learn to make more independent decisions [23,43]. Another study found a positive relationship between women and the degree of relational coordination [23].

### Strengths and limitations

One strength of this study is the collection of data on a wide array of care processes typical for specialized health-care settings. The inclusion of inter-professional teams from a broad range of clinical areas probably reduced the risk of selection bias. Furthermore, this inclusion increased the reliability and generalizability of the findings. The hierarchical statistical approach (which is appropriate when investigating associations of individual characteristics clustered at the team level) made false-positive findings (type I findings) less likely.

However, this study has several limitations. The cross-sectional design allowed us to identify associations and characteristics of inter-professional teams in specialized health-care settings but not determine causality.

The median response rate for surveys has declined slightly since 1975 [44]. In the present study, the response rate was acceptable (52%); however, we had limited information on individuals who did not return the survey, for example whether the majority were men or women. Consequently, an inclusion bias cannot be excluded. Further, the number of respondents in each care process is relatively low; results may therefore reflect a coincidental expression of the individual teams’ performance rather than cultural differences. However, more studies are needed to clarify these findings further.

Communication and relationships are believed to be different within and between professional groups [45]. By merging specific categories of professional groups in some analyses, we lost the possibility of identifying patterns or levels of responses specific to each of those groups. Further, although the Relational Coordination Survey showed satisfactory psychometric properties in earlier investigations [8,46], we cannot rule out measurement error or issues related to construct validity in the present study.

## Conclusion

This study represents the first exploration of inter-professional teamwork using the Relational Coordination Survey in a Norwegian context. The communication and relationship sub-scale scores were significantly higher within unique functional groups than between contrasting groups; this implies there is a need for inter-professional education programmes to enhance the understanding of health professionals’ roles and communication skills among team members. Our findings indicate that communication around specific groups of patients is better when team members use or develop a written clinical procedure in their clinical practice.

Future studies should be designed as longitudinal investigations. They should include outcomes at the patient and system level. They should also examine causal aspects of the communication and relationship skills of the Relational Coordination Survey to determine the quality of health-care delivery.
